# Long Covid: Untangling the Complex Syndrome and the Search for Therapeutics

**DOI:** 10.3390/v15010042

**Published:** 2022-12-22

**Authors:** Azizul Haque, Anudeep B. Pant

**Affiliations:** 1 One Medical Center Drive, Department of Microbiology and Immunology, Geisel School of Medicine at Dartmouth, Lebanon, NH 03756, USA; 2 New Orleans East Hospital, New Orleans, LA 70127, USA

**Keywords:** Long Covid, inflammation, autoimmunity, biomarkers, therapeutics

## Abstract

Long Covid can affect anyone who has previously had acute COVID-19. The root causes of this syndrome are still unknown, and no effective therapeutics are available. This complex syndrome, with a wide array of symptoms, is still evolving. Given the dire situation, it is important to identify the causes of Long Covid and the changes occurring within the immune system of affected patients to figure out how to treat it. The immune system intersects with the persistent viral fragments and blood clots that are implicated in this syndrome; understanding how these complex systems interact may help in untangling the puzzling physiopathology of Long Covid and identifying mitigation measures to provide patients some relief. In this paper, we discuss evidence-based findings and formulate hypotheses on the mechanisms underlying Long Covid’s physiopathology and propose potential therapeutic options.

## 1. Introduction

A sizable number of COVID-19 patients continue to experience symptoms after a period of acute illness; this condition was designated as Long Covid or post-acute sequelae of SARS-CoV-2 (PASC). A multi-system array of symptoms, such as breathlessness, muscle aches and fatigue, have been reported for months or even years after recovery from the initial infection. The WHO defines Long Covid as a “post-COVID-19 condition” that occurs if symptoms persist three months after infection and for which there is no alternative diagnosis [1]. This varies from the way other agencies define this condition; for example, the CDC defines this elusive syndrome as starting four weeks or more after the initial infection [2]. This condition can be devastating for some as the pathogenesis affects the function of multiple organs and causes cognitive impairment, thus, hindering patients from conducting normal daily activities. For this reason, the WHO is urging countries to make resources easily available at the community level for individuals suffering from Long Covid.

The WHO estimates that between 10% and 20% of those infected with the SARS-CoV-2 virus could endure persistent symptoms long after the acute infection has resolved [1,3]. A more recent study from Scotland indicates that nearly half of COVID-19 patients were still experiencing symptoms between 6 and 18 months following the initial infection [4]. The CDC estimates that nearly one in five American adults who have had COVID-19 are experiencing the effects of Long Covid [5]; of these roughly 34 million Americans, an estimated two to four million are unable to work due to complications from this syndrome [6]. Recent WHO data has revealed that 17 million people in Europe are thought to be living with Long Covid [7]. Data from the Office of National Statistics indicate that an estimated 2.3 million people living in private households in the UK (3.5% of the population) had self-reported experiencing Long Covid as of 4 June 2022 [8]. There are no clear data concerning the number of Long Covid cases globally at this particular time, and these numbers are expected to rise as COVID-19 becomes an endemic disease.

The large number of people suffering from post-acute COVID-19 symptoms and the absence of any effective treatment make this chronic condition concerning. In the UK, cases of Long Covid seen due to the Omicron BA.2 variant seem to be slightly fewer than previous variants [9,10]; however, Omicron was already the dominant variant at the time of this study and repeat or confirmatory studies are difficult to conduct with past variants. The risk factors that trigger the development of Long Covid are also not yet well defined. Recent studies proposed several risk factors that include gender, race/ethnicity, socioeconomic factors, smoking, obesity, and a wide range of comorbidities [8,11]. In other studies, additional risk factors described were age, hospital admission during acute infection, symptoms including dyspnea and chest pain, abnormal auscultation findings, and the presence of comorbidities such as asthma [12,13,14]. Furthermore, the presentation of this syndrome can vary depending on the risk factors and groups in question; for example, older individuals are already more likely to develop Long Covid; however, recent studies suggest that they have different persisting symptoms with more pronounced pulmonary impairment [15]. Robust population-based studies with appropriate control groups are required to identify biological risk factors specifically attributable to SARS-CoV-2 infection in both hospitalized and non-hospitalized individuals so that clinicians can better diagnose this evolving condition.

The mechanisms driving Long Covid are poorly understood. Recent studies have indicated that Long Covid is a multisystem disease that develops regardless of the initial disease severity [16]. Chronic systemic inflammation is frequently observed long after the clearance of acute COVID-19 infection [17]. This elevated prolonged inflammation, possibly resulting from organ damage [18], causes multiple complications in the bodies of individuals with Long Covid. Evidence also suggests that the risk to develop this condition is higher in women, the elderly, the economically disadvantaged and those who have existing physical and mental health conditions [4]. There is an urgent need to conduct further studies to better understand the risk factors and mechanisms underlying this physiopathology and to identify efficient treatment options.

## 2. Viral Persistence and its Role in the Development of Long Covid

A key question is whether lingering viruses or viral molecules are driving illness in Long Covid patients. NIH scientists have reported that tissues from COVID-19 patients, both mild and asymptomatic, showed the presence of viral RNA in various areas of the body, including in the brain, muscle, gut and lungs [19]. However, the study did not demonstrate a link between the persistent virus and Long COVID. Another question that needs to be answered is whether viruses found in Long Covid patients are capable of replicating.

A recent study in Austria found that patients with Long Covid symptoms harbored viral RNA or, in some cases, viral proteins in the gut [20]. The researchers seek to understand the behavior of the virus that lingers in the gut but have not been able to culture the virus from gut tissue in the lab. Investigating the presence of viruses in intestinal cells and the activity of immune cells that populate the region may provide answers as to whether these cells are in a heightened state of activation due to the presence of viral particles.

## 3. Symptoms and Underlying Potential Mechanisms

**Figure 1:**
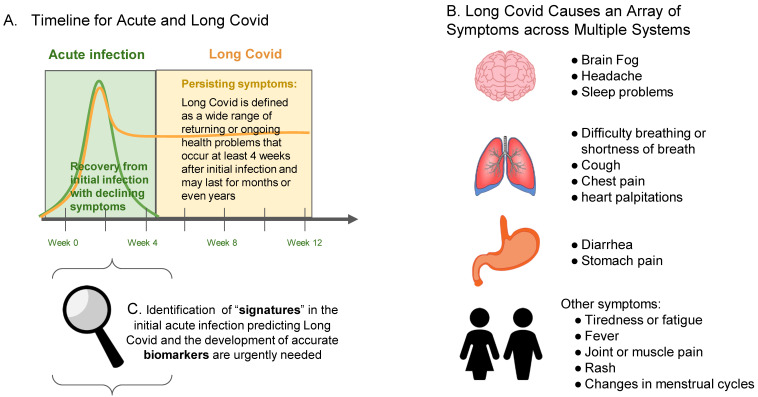
**Timeline and Symptoms of Long Covid. Panel A** demonstrates the approximate timeline for acute and Long Covid manifestation following infection with SARS-CoV-2 (note: the CDC states that four weeks after infections is when Long Covid conditions can first be identified); **Panel B** depicts the array of heterogenous symptoms caused by Long Covid across multiple systems; **Panel C** demonstrates the early time periods where accurate biomarkers identifying the onset of Long Covid could be most beneficial to attenuate disease progression.

Long-COVID is a multisystem disease, and its clinical spectrum comprises a wide range of symptoms. The symptoms are highly varied, ranging from lingering tiredness or coughs to diarrhea, rashes and disruptions to menstrual cycles [2] (Figure 1). In some cases, Long COVID can even manifest as depression and anxiety or cognitive problems such as “brain fog”. The evolving definition of the disease process and symptoms, in conjunction with the dearth of research, pose a significant challenge for clinicians looking to diagnose and provide patients with a clear prognosis. Evidence on why persistent symptoms occur is still limited and available studies are heterogeneous. Furthermore, there is no animal model of Long Covid, which limits researchers’ ability to untangle the complex syndrome.

**Figure 2:**
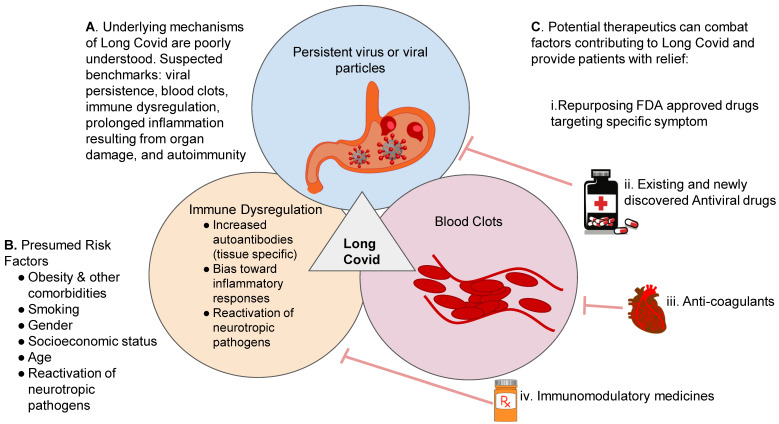
**Factors Contributing to Long Covid and Potential Treatment Options. Panel A** depicts suspected determinants contributing to Long Covid such as the presence of a virus or viral fragments after recovery from acute infection, chronic inflammation from organ damage, blood clotting dysfunction, and dysregulation of immune responses; **Panel B** demonstrates various risk factors including comorbidities, smoking, gender, age, and reactivation of latent pathogens; **Panel C** depicts potential therapeutic approaches, which include repurposing FDA approved drugs targeting specific symptom, the use of existing or newly discovered anti-viral drugs, anti-coagulant treatments and the use of immunomodulatory agents.

The available data suggests that Long Covid is likely to result from long-term organ damage, which occurs during the acute phase of the initial infection. Specific long-lasting inflammatory reactivity due to organ damage, central nervous system complications, gastrointestinal (GI) distress, auto-immunity, endothelial dysfunction, and coagulation dysregulation all have been implicated in the development of Long Covid pathogenesis (Figure 2). Studies indicate that myocarditis is seven times more likely to occur in patients with COVID-19 infection vs. patients who have been vaccinated [21].

There are presumably many factors that contribute to this syndrome. SARS-CoV-2-induced organ damage to one or multiple organs, persistent viral presence in certain tissues, re-activation of neurotrophic pathogens under conditions of a dysregulated immune response, SARS-CoV-2 interactions with host microbiome/virome communities, abnormal clotting/coagulation, altered brainstem/vagus nerve signaling, ongoing reactivity of primed immune cells and autoimmunity due to molecular mimicry between pathogen and host proteins have all been hypothesized to play a role [22] (Figure 2). Furthermore, it was also suggested that Long Covid symptoms may not be a direct result of the SARS-CoV-2 infection but may be the consequence of COVID-19-inflammation-induced EBV reactivation [23].

Blood clots have long been suspected to drive symptoms in Long Covid (Figure 2). In some Long Covid patients, the cells and tissues that control blood flow are damaged by the viral assault, and the blood’s tendency to clot is amplified. Minute blood clots, driven by a dysfunction in clotting protein, might be impairing the body’s circulation [24]. This may lead to reduced blood flow, causing devastating multisystem effects. The theory of persistent microclots was confirmed by a South African team who reported that such clots could linger in the blood of Long Covid patients [25]. The researchers reported finding signs of excessive blood clotting in 11 people with Long Covid but not in healthy people or another control group with type 2 diabetes.

Early in the pandemic, clinicians recognized blood clots as a signature of early, severe illness as many hospitalized patients had clots in their lungs, brain and elsewhere [26]. Pretorius et al. obtained preliminary data to suggest the number of microclots in blood correlates with the severity of some Long Covid symptoms, such as cognitive deficits [24]. One challenge facing microclot studies is that detecting them is a laborious process in a large number of patients.

Many suffer from fatigue and what is often called “brain fog.” Tiny clots in the brain could explain cognitive troubles, or clots may kill small fiber nerve cells and drive dysautonomia [27]. However, solid evidence that microclots cause Long Covid symptoms is still lacking, and the role of blood clotting in the development of Long Covid is debatable [28]. Of note, the incidence of deep vein thrombosis and pulmonary embolism in patients post hospital discharge is less than during hospitalization [29,30,31], which conflicts with the evidence presented in the aforementioned studies. Further studies are needed to investigate the role of clotting in Long Covid and to confirm if the process is occurring uniformly; however, in vivo studies are difficult to conduct. Researchers are investigating if apheresis, a therapy that filters blood, improves symptoms for a small cohort of Long Covid patients with microclots in their blood plasma. However, these same researchers share a note of caution that apheresis can “filter out lots of things in the blood”, which could also fuel symptoms [27].

Other neurological effects have been observed. A recent study described SARS-CoV-2’s ability to infect neurons and cause inflammation as early as one-week post-infection in the brains of rhesus macaques. Interestingly, the virus was observed to have infected neurons in the same regions of the brain that are known to be impacted by Alzheimer’s disease. These findings provide an important clue for researchers seeking to better understand the long-term neurological damage caused by Long Covid [32].

## 4. Long Covid in the Shadow of Altered Immune Responses and the Role of Autoimmunity

The initial acute infection might impact the host’s immune system and trigger chronic immune reactivities. A recent study suggests that pro inflammatory cytokines induce a self-sustaining inflammation signaling circuit that persists in Long Covid even after the virus has cleared [33]. Moreover, the persistent occult virus might also trigger changes after the acute infection, which manifest as chronic inflammatory responses [34]. It is possible that certain viral molecules present could elicit autoimmune responses in which the immune system attacks the body’s own tissues [35]. Further studies are needed to discern the causative relationship between autoimmune processes and lingering COVID-19 viruses. It is also possible that a SARS-CoV-2 infection can have long-term impact on gut microorganisms [36]. Although evidence is gradually accumulating that implicates virus/viral fragments, blood clots and altered immune responses, their links to Long Covid are still tenuous (Figure 2).

Recently, Su et al. carried out a longitudinal multi-omic study of >300 patients and presented evidence that some factors present at disease onset, such as pre-existing type 2 diabetes, latent Epstein–Barr virus (EBV) reactivation, circulating SARS-CoV-2 RNA fragments, as well as specific autoantibodies, associate with Long Covid [37]. The authors identified four different immune endotypes at 2–3 months post disease onset that differentially associate with Long Covid. Interestingly, the study found that the bystander activation of cytomegalovirus (CMV)-specific T cells during acute disease is associated with GI-Long Covid. Another study by Vijayakumar et al. shows persistent immunological and proteomic abnormalities in the lungs of patients with ongoing respiratory symptoms after COVID-19, with continuing activation of CD8^+^ T cells and elevated levels of proteins associated with apoptosis, tissue repair and epithelial damage [38].

It was observed that compared with uninfected controls and other infections such as EBV, CMV and HIV, SARS-CoV-2 infection is associated with the generation of a wide range of autoantibodies that can attack the tissues of infected subjects [39]. Some infected individuals show a high prevalence of autoantibodies against immunomodulatory proteins including cytokines, chemokines, complement components and cell-surface proteins [40]. Other autoantibodies are tissue-specific, including autoantibodies specific to blood vessels, the heart and the brain. Studies have described a tendency for some patients to develop over 15 separate types of autoantibodies and above 10 distinct autoimmune diseases [41].

The main mechanisms that may contribute to the development of autoimmunity in COVID-19 are the following: (1) hyper-activation of the immune system, (2) the induction of excessive neutrophil extracellular trap formation and (3) SARS-CoV-2 cross-reaction with self-components of the host. In fact, SARS-CoV-2 was shown to cross-react with gut, kidney, lung, heart and brain antigens, and SARS-CoV-2 proteins can share homology with some self-protein epitopes, leading to molecular mimicry paths [42]. Furthermore, under conditions of inflammation, other organisms of the microbiome/virome communities, which could vary widely between different patients, may also contribute to autoantibody production and cause the great variety in autoantibody reactivity [42]. This complex scenario could explain the significant percentage of clinical variations detected in patients with Long Covid.

Based on the current evidence, we hypothesize that inflammation and autoimmunity are the main drivers of Long Covid.

## 5. Long Covid in Children

Clinical data on Long COVID-19 in the pediatric population are limited. Insomnia, respiratory symptoms (including pain and chest tightness), nasal congestion, fatigue, muscle and joint pain and concentration difficulties were the most frequently reported symptoms [43]. Recently, Osmanov et al. observed that older age and allergic diseases were associated with a higher risk of persistent symptoms at follow-up [44]. This same study suggested that, at least in children, immunological mechanisms may be responsible for an increased risk of long-term consequences of infection. Recent data indicated that COVID-19 consequences may be linked with the mast cell activation syndrome [45] and the Th-2 biased immunological response in children with allergic diseases may be responsible for an increased risk of long-term consequences from the infection. A recent case study published by Italian physicians supports these observations [46]. A similar case study describes an adolescent who was diagnosed with mild COVID-19 disease initially; after the acute phase, at around 30 days, she developed persistent headache, chest pain, fatigue and tachycardia [47]. The study documents the first reported evidence of immune dysfunction and lung perfusion defects after mild COVID-19 in an adolescent. Once again, Long Covid detected in children was shown to be associated with allergic reactions [42].

## 6. Gender Disparity: A Puzzling Phenomenon of Long Covid

Studies demonstrate that women are more likely to report and experience symptoms of Long Covid [48]. This same study describes sex-disaggregated differences in Long Covid symptoms. Of note, women frequently pay more attention to their bodies, which often leads to a more rapid diagnostic and therapeutic intervention in general. Females have both innate and acquired immunological responses stronger than males, and both genes and hormones are involved in this sex difference [49,50]. These sex-based immunological differences contribute to variations in the incidence of autoimmune diseases, susceptibility to malignancies and infectious diseases and probably represent the major cause of the female prevalence of Long Covid in adults. Sex-based differences were also observed in Lyme disease, where the occult bacteria or bacteria fragments generate a more robust cytokine response in women than men [51]. Of note, no significant difference was reported to date between the youngest male and female patients [44], which supports the hypothesis that sex hormones and their immunomodulating activity could play a role in adult Long Covid patients [52]. Females are more prone to developing autoimmune diseases than males [53]; if Long Covid manifests as an autoimmune disorder, this could explain why this syndrome disproportionately affects women.

## 7. Biomarkers

There is an urgent need for the development of lab tests to evaluate the risks of Long Covid and to develop new therapies to tackle this form of post-COVID-19 syndrome. Identifying a biological molecule in the blood that correlates with the onset of this syndrome would be of paramount importance to tackle this challenge (Figure 1).

Recent studies found significantly higher levels of SARS-CoV-2 nucleocapsid protein and spike protein in blood plasma samples collected between 6 and 12 weeks after diagnosis from patients infected with COVID-19 who had neuropsychiatric symptoms [54]. They also found significant differences in levels of several mitochondrial proteins between Long Covid patients with and without neuropsychiatric symptoms, pointing to alterations in mitochondrial function within neurons [54]. Further studies are required to assess whether mitochondrial proteins could be used as biomarkers for Long Covid.

The spike protein, S1 subunit or nucleocapsid was found to be present in the blood of 65% of the Long Covid patients tested, up to 12 months after their initial COVID-19 infection [55]. Out of the three SARS-CoV-2 antigens, the spike protein was the most common, having been detected in 60% of Long Covid patients. Conversely, no spike protein was detected in any of the patients with a typical COVID-19 infection. The researchers believe that the presence of the SARS-CoV-2 spike protein in Long Covid patients for up to 12 months suggests the presence of an active persistent SARS-CoV-2 viral reservoir (Figure 2). The presence of the spike protein in the majority of Long Covid patients suggests that the spike protein could potentially be used as a biomarker for Long Covid. Before using the spike protein as a diagnostic tool, however, researchers will need to conduct further studies to confirm whether detection correlates with Long Covid symptoms and the accurate percentage of Long Covid patients harboring the spike protein.

Other studies have pointed to the gut as a possible reservoir. A group from Stanford has found that 4% of Individuals with mild to moderate COVID-19 continued shedding viral RNA in their stools seven months after the COVID-19 diagnosis [56]. Individuals with detectable viral RNA in their stools also reported ongoing gastrointestinal symptoms such as abdominal pain, nausea and vomiting.

Biomarker findings based on a single time-point may not predict the actual stage of Long Covid, a complex syndrome. Patients that are monitored at differing time points may show changes in symptoms and immune responses in Long Covid. We propose that the biomarkers, including immunological markers, that can detect the onset of Long Covid and can identify the immunological and biological parameters correlating with symptoms would be of paramount importance for better management and treatment of patients (Figure 1).

## 8. Treatments

Clinicians are faced with the challenge to identify treatments that ease or reverse the abnormalities caused by Long Covid and help patients feel better. Some recent research suggests the risk of developing Long Covid is somewhat lower for vaccinated people [57,58]; however, it remains uncertain if vaccination can curtail this syndrome. More research is needed to identify existing drugs and/or discover novel therapeutics that could ameliorate symptoms of Long Covid.

Each of the reactivities seen in Long Covid patients suggests a route to relief; drugs that suppress the immune system could regulate an altered immune response; antiviral drugs may target and clear persistent occult reservoirs of SARS-CoV-2; microclots may be combated by anti-coagulant therapies (Figure 2). Clinicians warn that using anticoagulants as a treatment carries the risk of severe bleeding and any such treatment should be only given under the careful guidance of a clinician. Of note, most societies only recommend treatment with post-hospital discharge prophylaxis for high-risk patients [59].

Paxlovid and molnupiravir are the first oral medications to be approved for treating mild to moderate COVID-19 and are proven to reduce the mortality and hospitalization rates in patients with COVID-19 [60]. Molnupiravir (Lagevrio) is made by Merck, and ritonavir (Paxlovid) is manufactured by Pfizer. Another drug, remdesivir (Veklury), made by Gilead Sciences, has been used to treat hospitalized COVID-19 patients since the early days of the pandemic and was recently approved as an outpatient therapy for people at high risk for developing COVID-19 complications. Of note, there are currently no clinical trials aimed to assess the impact of these existing antiviral therapies on Long Covid, though a recent preclinical study found that molnupiravir attenuated chronic Long Covid manifestations [61].

While antiviral treatments remain a powerful tool to combat COVID-19, adverse drug interactions need to be carefully monitored. Recently, the FDA has developed a checklist to assist clinicians with evaluating potential drug interactions and other patient related risk factors before prescribing Paxolovid [62]. Given safely, early diagnosis and treatment with oral antivirals are beneficial for COVID-19 prognosis and may prove to be useful in preventing Long Covid as well. However, as testing rates drop off and in-home testing becomes the norm, pre-symptomatic detection of the virus may not be feasible.

Other trials are aimed at impacting dysregulated immune responses. The NIH is testing several familiar immunomodulatory drugs, such as Infliximab, which is approved for the treatment of Crohn’s disease [63,64]. Other trials are using drugs that have shown some success in treating severe acute COVID-19, including steroids and other immunosuppressants [65]. Reports of observational and case studies suggest that antihistamines show some relief [66,67]. These preliminary findings support a case for additional randomized trials of antihistamines in Long Covid. Additionally, an experimental drug called RSLV-132 is being investigated to test the efficacy and safety of the drug in subjects with Long Covid [68]. RSLV-132 is an enzymatically active ribonuclease designed to digest the ribonucleic acid contained in autoantibodies and immune complexes and, thereby, render them biologically inert. This treatment approach is directed at taming inflammation in people with Long Covid. High serum levels of IL-17 and IL-2 and low levels of IL-4 and IL-10 appear to constitute a cytokine profile **in Long Covid patients** [69]. **Further investigations are necessary to provide evidence whether they** could be useful targets for treatment and **designing** prevention strategies.

For a syndrome such as Long Covid, which seems to have multiple pathogenic mechanisms and symptom subsets, we hypothesize that recombinational therapy may ease Long Covid symptoms. In the absence of effective drugs in the early days of the Ebola epidemic, we proposed that repurposed drugs that are FDA-approved might be tested [70]. Currently approved drugs that are known to work against other diseases with similar mechanisms and symptoms might be considered for use in Long Covid (Figure 2). Some of the immunomodulatory drugs used in Multiple Sclerosis could be repurposed to manage COVID-19-associated immune dysregulation. Clinical trials are ongoing to evaluate various Multiple Sclerosis therapies [71], and masitinib was recently highlighted as a potent Coronavirus inhibitor [72].

If we were to use a new treatment for Long Covid, it should be based on a robust clinical trial that would include a placebo group. In the coming months, key trials could yield results for drugs that modulate the immune system and target blood clots or persistent viral reservoirs or fragments. There is a fair amount of funding for discovering therapies against Long Covid so relief for patients suffering from this syndrome is on the horizon.

A note of caution is that there is a risk of viral drug resistance in the case of monotherapies if they target only one part of the virus. This underscores the need to develop better antivirals aimed at diverse targets, or combinations thereof, that can be combined into a single treatment to target the virus in multiple ways. We hypothesize that a breakthrough therapeutic would be a broad-spectrum drug capable of recognizing targets that are common to entire families of viruses, including variants. This type of broad-spectrum drug and a universal vaccine will be our best tools in combating current and future pandemics and their associated syndromes, such as Long Covid.

## 9. Conclusions

The scientific evidence on why persistent symptoms occur in some COVID-19 patients after recovery from the initial acute infection is still limited and treatment options are limited. Further studies are needed to better understand the mechanisms underlying Long Covid and to identify targets for efficient therapeutics. As the estimates of patients suffering from Long Covid continue to balloon in size, it is becoming increasingly important to define the symptoms associated with the disease so clinicians can diagnose and treat patients more effectively. It is important to determine the “signature” of Long Covid in the early phases of COVID-19, if any. The biomarkers for early identification of Long Covid would go a long way toward providing treatment and better disease management. Researchers need to focus on developing better vaccines that are able to tackle the currently circulating virus earlier in the acute infection or the virus that is hiding in the body in the later phase of infection. Finally, discovering novel antiviral drugs or repurposing immunomodulatory medications that will alleviate the patient’s symptoms is of paramount importance to tackle this emergent public health issue.

Until clear treatment strategies are identified, public health officials could focus their energies on providing support to patients as they recover from this debilitating syndrome. Support groups and tailored rehabilitation may provide much-needed relief to patients looking to resume their normal daily routines and reduce long-term disability. As the estimates for this condition continue to climb, public health officials will need to implement every resource available to combat this emerging public health concern.
